# *Zingiber chengii* (Zingiberaceae), a new species from Taiwan

**DOI:** 10.3897/phytokeys.139.37294

**Published:** 2020-01-15

**Authors:** Chiu-Mei Wang, Yuan-Chien Lin, Yen-Hsueh Tseng

**Affiliations:** 1 Department of Biology, National Museum of Natural Science, 1 Guanchien Rd., Taichung 40453, Taiwan National Museum of Natural Science Taichung Taiwan; 2 Department of Forestry, National Chung Hsing University, 145 Hsing-Ta Rd., Taichung County 40227 Taiwan National Chung Hsing University Taichung Taiwan

**Keywords:** northern Taiwan, riverside, rock cliff, *
Zingiber
*

## Abstract

In this article, we describe a new species, *Zingiber chengii* Y.H. Tseng, C.M. Wang & Y.C. Lin, discovered on a rock cliff of Youluo riverside in northern Taiwan. This species is easily distinguished from other known congeners by its grass-like leaves, spikes composed of a few sterile bracts, and seeds one-third enveloped by the aril. Color illustrations, line drawings, and a key to species of *Zingiber* in Taiwan are provided as well as comparative morphology in relation to its allied species, geographical distribution, and conservation status.

## Introduction

*Zingiber* Mill (Zingiberaceae) comprises approximately 100−150 species, with its center of diversity in Southeast Asia (Wang 2000, Wu and Larsen 2000, Theerakulpisut et al. 2012). *Zingiber* spp. are mostly perennial herbs, characterized by a pulvinus leaf base (a swollen part of the petiole) and a horn-shaped anther crest embracing the upper part of the style (Bai et al. 2015a). Several species in this genus are known to be widely cultivated in tropical Asia, such as *Z. officinale* Roscoe and *Z. zerumbet* (L.) Sm., and carry great economic value (Wang 2000). The genus *Zingiber* is divided into Z. sect. Zingiber, sect. Dymczewiczia (Horan.) Benth., sect. Pleuranthesis Benth., and sect. Cryptanthium Horan. based on the position of the inflorescence (Schumann 1904). Additionally, species of the sections *Zingiber* and *Dymczewiczia* have spherical pollen grains with cerebroid sculpturing, while those belonging to the sect. Cryptanthium have ellipsoidal pollen grains with spiro-striate sculpturing (Theilade et al. 1993).

Three native species of *Zingiber* have been recognized by Wang (2000) in Taiwan, i.e. *Z. kawagoii* Hayata, *Z. oligophyllum* K.Schum and the insufficiently studied *Z. pleiostachyum* K. Schum. Subsequently, *Z. shuanglongense* C.L.Yeh & S.W.Chung were described from central to southern Taiwan (Yeh et al. 2012). All four Taiwanese species belong to sect. Cryptanthium.

Recently, we discovered an unknown *Zingiber* in northern Taiwan belonging to the Z. sect. Cryptanthium, as indicated by the radical inflorescences with a procumbent peduncle. Here, we describe this new species of *Zingiber* and evaluate its conservation rank.

## Materials and methods

An unknown species of *Zingiber* was found abundant on a rock cliff of Youluo riverside, where more than 100 individuals were observed in an area of ca. 400 m^2^ (24.694, 121.184). In addition, more than 50 individuals were discovered in similar habitat along the same riverside (24.695, 121.220). Morphological measurements were made from both herbarium and spirit samples by a ruler and digital calipers. For morphological descriptions, the terminology used by Beentje (2012) and Leong-Škorničková et al. (2014) was followed.

Protologues of *Zingiber* spp. and herbarium specimens were examined, including type specimens deposited in HAST, IBSC, NTNU, TAI, TAIF, TCF, TI, TNM, and PPI, in addition to specimens at K, UPS, and US, which were available as images. Considering the similarity of the newly collected species and *Z. tenuifolium* L. Bai, Škorničk. & N.H. Xia, we also compared the Taiwanese species with *Z. tenuifolium*, as described by Bai et al. (2015b).

The conservation rank for the new species was evaluated according to IUCN (2017). Pollen grains for scanning microscope examination (voucher: *Z. chengii* Hsinchu County, Jianshih Township, *Y.C.Lin 1116* & *1148*, TCF) were prepared following Halbritter (1998): anthers were treated with DMP (2, 2-Dimethoxypropane) for 30 minutes and transferred to acetone for 30 minutes and critical-point dried. The material was mounted on a stub and sputter coated with gold (Quorum SC7620) and examined using a Hitachi S-3400N microscope.

A distribution map was generated by using QGIS ver. 3.4 from package of Lin (2018).

## Taxonomic treatment

### Zingiber chengii

Taxon classificationPlantaeZingiberalesZingiberaceae

Y.H.Tseng, C.M.Wang, & Y.C.Lin sp. nov.

2AEBBFFC-E079-5F09-8972-44F53B242E43

urn:lsid:ipni.org:names:77204420-1

**Figure 1.**
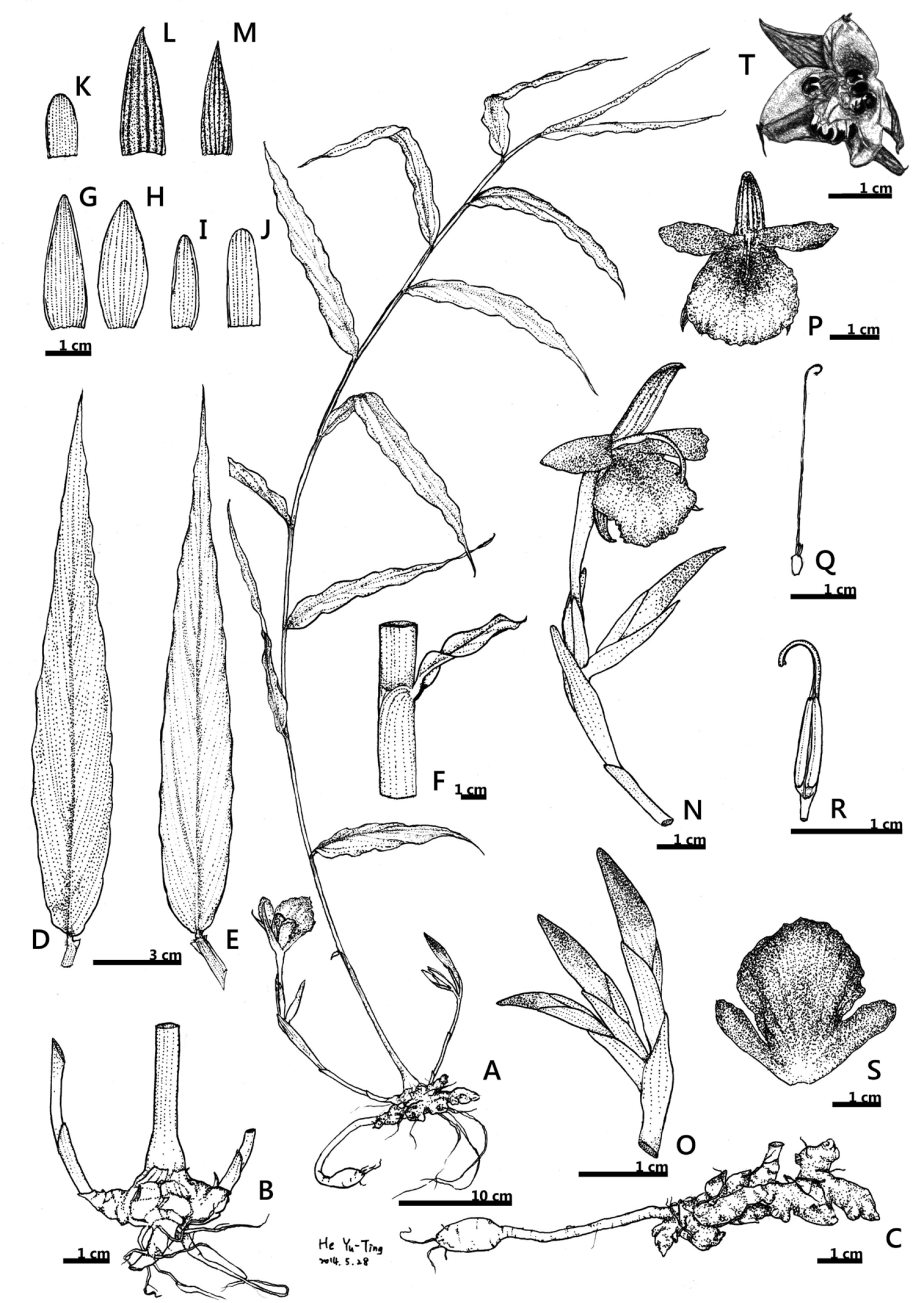
Line drawings of *Zingiber chengii* Y.H.Tseng, C.M.Wang & Y.C.Lin , sp. nov. **A** habit **B** base of plant **C** rhizome **D−E** leaf adaxial and abaxial surface **F** ligulate **G−K** bracts and bracteoles **L** dorsal corolla lobe **M** lateral corolla lobe **N−O** inflorescences **P** flower **Q** pistil **R** stamen and anther crest **S** labellum with basally connate lateral staminodes **T** fruit.

**Figure 2.**
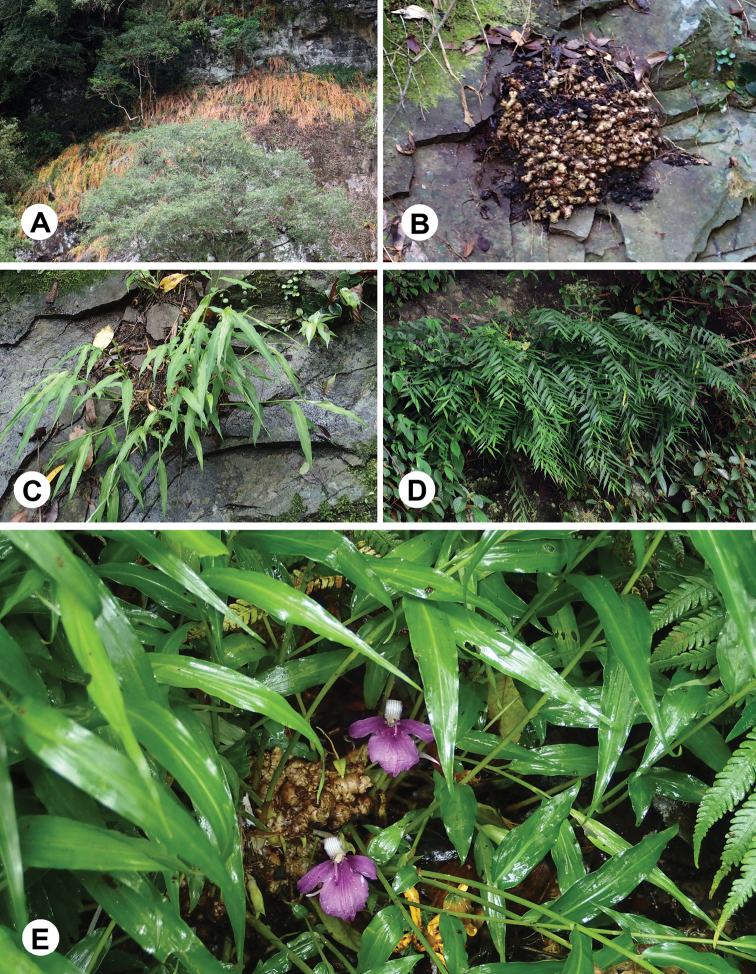
Phenologic phases of *Zingiber chengii* Y.H.Tseng, C.M.Wang & Y.C.Lin, sp. nov. **A** withering period **B** dormant period (rhizome) **C** growth period **D** mature period **E** flowering period.

**Figure 3.**
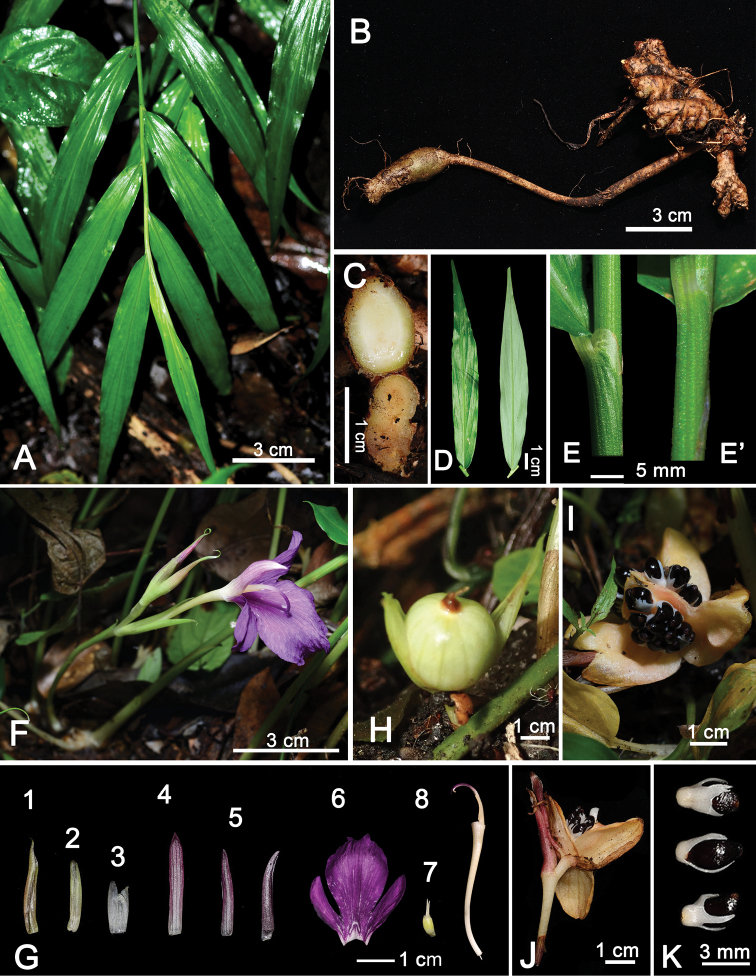
*Zingiber chengii* Y.H.Tseng, C.M.Wang & Y.C.Lin, sp. nov. **A** habit **B** rhizome **C** the cross-section of rhizome **D** leaf blade **E** ligule and sheath (side view) **E**’ sheath (front view) **F** inflorescence **G** flower dissection **1** fertile bracts **2** Bracteole **3** calyx **4** dorsal corolla lobe **5** lateral corolla lobes **6** Labellum with basally connate lateral staminodes **7** ovary **8** floral tube with stamen and stigma (side view) **H−J** fruit **K** seeds.

**Figure 4.**
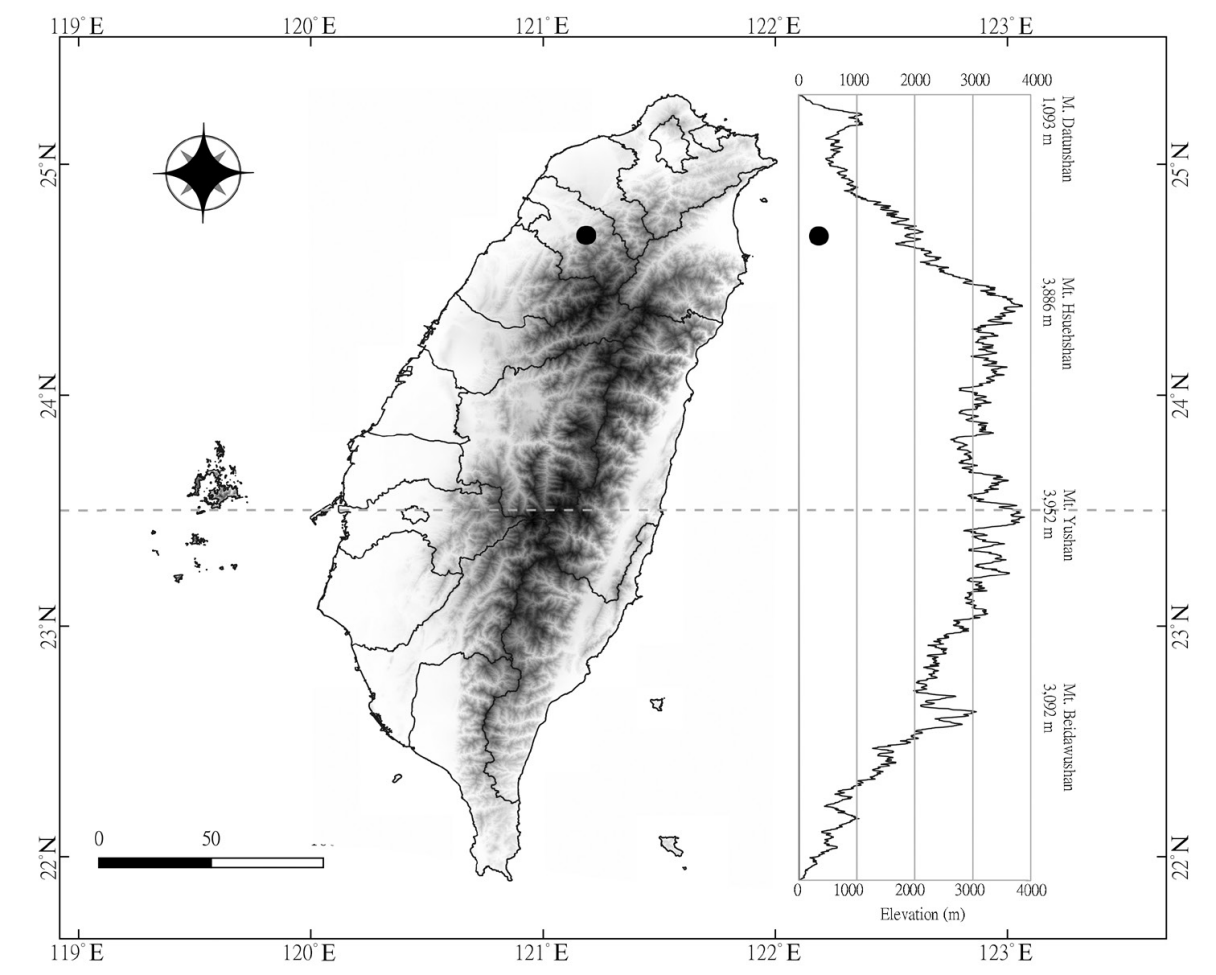
Distribution map of *Zingiber chengii* Y.H.Tseng, C.M.Wang & Y.C.Lin, sp. nov.

**Figure 5.**
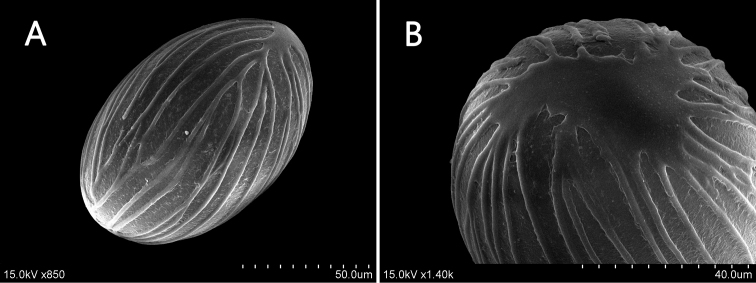
Pollen morphology of *Zingiber chengii* Y.H.Tseng, C.M.Wang & Y.C.Lin, sp. nov. **A** equatorial view **B** polar view.

Figs 1 , 2 , 3 , 4 , 5

#### Diagnosis.

*Zingiber chengii* sp. nov. is morphologically similar to its Taiwanese congeners. However, the new species can be distinguished from them by its deciduous leafy shoots while those of *Z. kawagoii*, *Z. oligophyllum* and *Z. shuanglongense* are evergreen; *Z. chengii* has narrow lanceolate to linear leaves, whereas *Z. kawagoi* and *Z. shuanglongense* have ovate to lanceolate ones; except *Z. oligophyllum*, which has yellow flowers, all native species of Taiwan have reddish-purple flowers; each spike of *Z. chengii* bears 1−3 flowers, whereas spikes of *Z. kawagoi* and *Z. shuanglongense* bear 8−11 and 4−10 flowers, respectively; *Zingiber chengii* rarely has sterile bracts, whereas *Z. kawagoii* and *Z. shuanglongense* have apparent sterile bracts; *Zingiber chengii* has ovoid fruit, whereas *Z. kawagoii* and *Z. shuanglongense* has ellipsoidal one. Both *Z. kawagoii* and *Z. shuanglongense* are almost enveloped by the aril, whereas *Z. chengii* is one-third enveloped by the aril (Table 1).

**Table 1.** Morphological characters of *Zingiber chengii, Z. kawagoii, Z. shuanglongense*, and *Z. tenuifolium*.

**Character**	***Z. chengii***	***Z. kawagoii***	***Z. shuanglongense***	***Z. tenuifolium***
Rhizome	yellowish	yellow to greenish yellow	dark violet internally	yellow to greenish yellow
Leafy shoots	spreading to weakly arching, 11–15 leaves	erect, 6–21 leaves	erect, or slightly inclined, 7–21 leaves	spreading to weakly arching, 13–23 leaves
Lamina shape	linear-lanceolate to lanceolate, 9–15 × 1.5–2.5 cm	narrowly oblong to lanceolate, 12–29 × 3–8.5 cm	narrowly oblong to lanceolate, 12–23 × 2–7 cm	linear to narrowly ovate, 18–23 × 1.5–3.0 cm
Lamina length: width ratio	ca. 6	ca. 3.8	ca. 3.7	ca. 10
Flower number of each spike	1−3	8−11	4−10	unknown
Floral tube	extending at least 15 mm beyond the bract	extending at least 10 mm beyond the bract	extending at least 10 mm beyond the bract	extending only 2 mm beyond the bract
Color of corolla tube	cream-white	yellowish	cream-white	white with slight pink
Labellum	widely obovate, 21–33 × 29–19 mm, margin crisped, apex retuse or entire	obovate to oblong, 15–20 × 5–10 mm, apex retuse or entire or acuminate	broadly ovate or obovate, 24–34 × 15–16 mm, apex retuse or cleft	subrhombic to ovate, 24–28 × 13–17 mm, margin crisped, apex acuminate obtuse or shortly incised
Lateral staminodes	narrowly oblong, 18–24 × 4–7 mm, basal 1/3 to 1/4 connate to labellum, apex acute or obtuse	oblong, 14–18 × 5–6 mm, basal 1/2 to 2/3 connate to labellum, apex acute or obtuse	narrowly oblong, 15–29 × 3–6 mm, basal 1/3 to 1/4 connate to labellum, apex acute or obtuse	narrowly ovate, 13–18 × 3–5.5 mm, basal 1/3 to 1/2 connate to labellum, apex acute or obtuse
Color of labellum and lateral staminodes	violet, scattered with cream-white patches at base	red or deep violet, yellowish at base	violet, scattered with cream-white patches at base	deep violet with cream-white patch at base
Fruit shape	ovate	elliptic	elliptic	unknown
Seed enveloped by the aril	1/3	3/4	3/4	unknown

Compared with the images of the syntype of *Z. pleiostachyum*, *Z. chengii* has much narrower lamina, with a length: width ratio of ca. 6 (vs. ca. 3.8 in *Z. pleiostachyum*) and rarely has sterile bracts. *Zingiber chengii* is similar to *Z. tenuifolium* L. Bai endemic to Yunnan (Bai et al. 2015b), but the number of blades per leafy shoot of *Z. chengii* is about 11–15 vs. 13–23 in *Z. tenuifolium*. The two species can also be distinguished by the length to width ratio of the lamina, which is ca. 6 in *Z. chengii* vs. ca. 10 in *Z. tenuifolium*. *Zingiber tenuifolium* also has apparent sterile bracts while these are rare in *Z. chengii*. These comparisons indicate that *Z. chengii* is clearly different from other known similar congeners, therefore we treat *Z. chengii* as a new species in Taiwan. Also, *Z. chengii* has ellipsoidal pollen grains with spiro-striate sculpturing (Fig. 5), and the inflorescence borne on a radical, procumbent peduncle (Fig. 1A, 2E, 3F). These characters indicate that this new species belongs to sect. Cryptanthium.

#### Type.

TAIWAN. Hsinchu County, Jianshih township, elevation ca. 320 m, 23 May 2014. *Yen Hsueh Tseng 5614* (Holotype: TCF).

#### Description.

Perennial rhizomatous herbs, 40–70 cm tall. Rhizomes fleshy, compacted, sympodial, densely branched, 0.8–1.4 cm in diameter, surface brown, center light yellow; root tubers terete, distantly from the rhizomes, ca. 3.8 × 1.2 cm, surface brownish green. Leafy shoots erect, 1–16 per plant, forming dense clumps, spreading, each shoot comprising 11–15 well-developed leaves at anthesis. Leaves deciduous, simple, distichous; ligules ca. 2 mm long, bilobed, membranaceous, pale green, auriculate; petiole 2.0−3.0 mm long, adnate to lamina by a pulvinus; lamina linear-lanceolate to lanceolate, 9−15 × 1.5−2.5 cm, length:width ratio 5.1−6.6, adaxial surface green, glabrous, abaxial surface pale green, pubescent along the midrib, base cuneate obtuse, apex acuminate, margin entire, conspicuously undulate, chartaceous. Spike 1–2 per plant, arising from rhizomes; peduncles 2.5–6.2 cm long, ascending, glabrous; spike narrowly oblong, ca. 10.5–12.5 × 2.0–3.0 cm, each with 1–3 flowers; fertile bracts yellowish green, one-flowered, lanceolate, 2.5–3.0 × 0.6–0.8 cm, usually red tinged, usually involute on both sides, apex acute to attenuate; bracteole lanceolate, 1.8–2.8 × 0.6–0.8 cm, translucent green with slight red tinge, apex acute. Flowers ca. 7.0–9.0 cm long, exerting much beyond the bracts; calyx tubular, membranaceous, ca. 7 mm long, with unilateral incision, translucent. Corolla tube slender, ca. 3.5-cm long, cream-white, glabrous externally and internally; dorsal corolla lobe lanceolate, ca. 2.7 × 0.7 cm, purple, apex acuminate; lateral corolla lobes lanceolate, ca. 2.5 × 0.7 cm, purple, apex acuminate; labellum widely obovate, ca. 3.0 × 2.5 cm, purple, apex retuse or entire, scattered with cream-white patches at base; lateral staminodes narrowly oblong, ca. 2.0 × 0.5 cm, connate to labellum at ca. basal 1/3 to 1/4, purple. Stamen one; filament short; anther connective tissue cream-white, elongated appendage of a wrapped style; anther thecae two, ca. 1 cm long, longitudinal dehiscense, pollen light yellow; anther crest beak-shaped, ca. 1.5-cm long when stretched, purple, apex entire. Style filiform, white, ca. 5.5-cm long, extending to the end of anther crest; stigma white, ciliate. Ovary cylindrical, trilocular, ca. 6.0 × 3.0 mm, yellowish green, glabrous; epigynous glands two, narrowly conical, ca. 6-mm long, pale yellow, apices sharp. Capsule ovate, dehiscence loculicidally ca. 1.5 × 1.3 cm, usually as long as the persistent bract, pericarp yellowish cream or orange-red inside. Seed ellipsoid, ca. 4.0 mm × 2.0 mm, enveloped by the aril. Aril white, deep denticulate at apex, enveloping 1/3^rd^ of the length of the seeds. Pollen grains ellipsoidal, 103.16–112.01 × 68.73–81.73 μm with P/E ratio 1.32–1.56, surface inaperturate and with spiro-striate sculpturing (Fig. 5).

#### Phenology.

Flowering between May and July, and fruiting between July and September. Growth and reproduction period between March and September, withering from September to November, and dormant period between December and February (Fig. 2).

#### Distribution and habitat.

Endemic species of Taiwan. Based on the geographical climatic regions and vegetation zones (Su 1984, 1985), *Z. chengii* is distributed only in the northwest inland region, moist areas of cloud forests of the *Machilus*–*Castanopsis* forest zone at an altitude of 530 m, and is found only on the rock cliff of Yuluo riverside (Hsinchu County) in northern Taiwan (Fig. 4). Common companion species are *Arundo formosana* Hack. (Poaceae), *Sedum actinocarpum* Yamam. (Crassulaceae), *Rhaphidophora hongkongensis* Schott (Araceae), *Pothos chinensis* (Raf.) Merr. (Araceae), *Pilea plataniflora* C.H.Wright (Urticaceae), and *Pyrrosia lingua* (Thunb.) Farw. (Polypodiaceae). Sometimes, *Z. kawagoii* is found nearby; however, no potential hybrid individual has been observed.

#### Chinese name.

Hsia-yeh-chiang (狹葉薑).

#### Etymology.

The species epithet “*chengii*” was given in honor of Mr. Yuen-Chun Cheng (鄭元春) who first discovered the new species.

#### Conservation status.

*Zingiber chengii* has been abundant on the rock cliff of Youluo riverside, where more than 100 individuals have been observed in an area of ca. 400 m^2^, since 2014. However, its population gradually decreased due to disturbances by visitors. Additional specimens were discovered along the Yuluo riverside in similar riverine habitats. These areas are difficult to approach due to the presence of hazardous rivers and cliffs. We categorize the new species as Endangered (EN B1; C2a(i)) following IUCN (2017).

#### Additional specimens examined.

*Zingiber chengii*: TAIWAN. Hsinchu County, Hengshan Township (24.694, 121.184), 23 May 2014. *Yen Hsueh Tseng 5615* (TCF); same loc., 29 May 2017. *Chao 4471* (TAIF); same loc., 25 July 2014. *Chiu-Mei Wang & Ching-Yao Li 16051* (TNM); same loc., 7 June 2015. *Y.C. Lin 1148* (TCF); Bilin Bridge (24.695, 121.220), 1 July 2015. *Y.C. Lin 1355* (TCF).

*Zingiber shuanglongense*: TAIWAN. Nantou County, Sinyi Township, Shuanglung Logging Trail, *Y.C.Lin 1294* (TCF); Jenlun Logging Road, *Y.C.Lin 1306* (TCF); Chiayi County, Jhuci Township, Mt. Dadungshan backbend (huitouwan), *Y.C.Lin 1292* (TCF); Kaohsiung City, Taoyuan District, Tengchih, *Y.C.Lin 1256* (TCF); Jiasian District, Mt. Paiyun, *Y.C.Lin 1319* (TCF).

*Zingiber kawagoii*: TAIWAN. New Taipei City, Shiding District, Mt. Erhkeshan, *Y.C.Lin 1066* (TCF); Nantou County, Jiji Township, Mt. Chichidashan, *Y.C. Lin 1290* (TCF); Chiayi County, Alishan Township, Lungtou, *Y.C. Lin 1278* (TCF), Mihu trail, *Y.C.Lin 1151* (TCF); Kaohsiung City, Maolin District, Shanping, *Y.C. Lin 985* (TCF); Pingtung County, Shizi Township, Shuangliu Forest Recreation Area, *Y.C. Lin 1303* (TCF).

*Zingiber pleiostachyum*: TAIWAN. Syntype: Pingtung County, Bankinsing mountains, *A. Henry 147* (K & UPS) & *1659* (K).

##### Identification key to the species of *Zingiber* in Taiwan

**Table:** 

1	Ligules reduced, weakly bilobed; labellum yellowish	***Z. oligophyllum***
–	Ligules bilobed; labellum violet or reddish	**2**
2	Spike rarely has sterile bracts; capsule ovate; 1/3^rd^ of seed enveloped by the aril	***Z. chengii***
–	Spike has sterile bracts; capsule elliptic; 3/4^th^ of seed enveloped by the aril	**3**
3	Corolla tube yellowish; 1/2 to 1/3 of lateral staminodes connate to labellum; the capsule length is 1/2−2/3 of the persistent bract	***Z. kawagoii***
–	Corolla tube cream-white; 1/3 to 1/4 of lateral staminodes connate to labellum; capsule equal to or longer than the persistent bract	***Z. shuanglongense***

## Supplementary Material

XML Treatment for Zingiber chengii
